# Longitudinal Analysis of Patient Specific Predictors for Mortality in Sickle Cell Disease

**DOI:** 10.1371/journal.pone.0164743

**Published:** 2016-10-20

**Authors:** Susanna A. Curtis, Neeraja Danda, Zipora Etzion, Hillel W. Cohen, Henny H. Billett

**Affiliations:** 1 Department of Medical Oncology and Hematology, Yale New Haven Medical Center, New Haven, CT, 06510, United States of America; 2 Division of Hematology, Department of Medicine and Oncology, Montefiore Medical Center, Albert Einstein College of Medicine, Bronx, NY, 10467, United States of America; 3 Department of Epidemiology and Population Health, Albert Einstein College of Medicine, Bronx, NY, 10467, United States of America; Morehouse School of Medicine, UNITED STATES

## Abstract

**Introduction:**

White Blood Cell (WBC) count, %HbF, and serum creatinine (Cr), have been identified as markers for increased mortality in sickle cell anemia (SCA) but no studies have examined the significance of longitudinal rate of change in these or other biomarkers for SCA individuals.

**Methods:**

Clinical, demographic and laboratory data from SCA patients seen in 2002 by our hospital system were obtained. Those who were still followed in 2012 (survival cohort) were compared to those who had died in the interim (mortality cohort). Patients lost to follow-up were excluded. Age adjusted multivariable Cox proportional hazards models were constructed to assess hazard ratios of mortality risk associated with the direction and degree of change for each variable.

**Results:**

359 SCA patients were identified. Baseline higher levels of WBC, serum creatinine and hospital admissions were associated with increased mortality, as were alkaline phosphatase and aspartate aminotransaminase levels. Lower baseline levels of %HbF were also associated with increased mortality. When longitudinal rates of change for individuals were assessed, increases in Hb or WBC over patient baseline values were associated with greater mortality risk (HR 1.54, p = 0.02 and HR 1.16, p = 0.01 with negative predictive values of 87.8 and 94.4 respectively), while increasing ED use was associated with decreased mortality (HR 0.84, p = 0.01). We did not detect any increased mortality risk for longitudinal changes in annual clinic visits or admissions, creatinine or %HbF.

**Conclusions:**

Although initial steady state observations can help predict survival in SCA, the longitudinal course of a patient may give additional prognostic information.

## Introduction

The past half-century has seen ever-improving survival among those with sickle cell anemia (SCA). In 1973 it was estimated that the median age of survival was 14.3 years for both sexes, however in 2010 it was estimated that 93.4% of all those with sickle cell disease (SCD) would survive to age 18, and a study in 2014 estimated a median survival of 58 years for both sexes with SCA [1–3].

There is, however, significant variation in longevity among those with sickle cell anemia. Finding markers that may be useful in mortality risk stratification is important in light of increasing use of stem cell transplant as a curative procedure for SCD [4, 5]. Previous studies have identified renal failure, acute chest syndrome, increased episodes of pain crisis, low hemoglobin F (HbF), low hemoglobin (Hb) and elevated white blood cell count (WBC) as associated with early mortality [1, 2, 6–8]. These studies examined static values and did not evaluate whether the degree of change in clinical and laboratory parameters over time—a longitudinal analysis—might also be pertinent in assessing risk factors. If we can identify biomarkers that allow a longitudinal approach to stratification of severity of disease, then we could have a therapeutic paradigm of “watchful waiting” with appropriate intervention. We sought to examine whether there was an additional benefit to assessing the longitudinal trajectory of some parameters over the assessment of their initial baseline levels.

## Methods

### Study Population

Our study was submitted to and approved by the Montefiore Medical Center Institutional Review Board. No informed consent was obtained as the data was analyzed anonymously. We used our electronic medical records system and Clinical Looking Glass (CLG), a user-friendly interactive software application developed at Montefiore Medical Center to evaluate health care quality, effectiveness, and efficiency. The system integrates clinic and administrative data sets allowing clinicians to extract cross-sectional and longitudinal data suitable for epidemiological analyses. Patients with sickle cell anemia (SCA, here defined as SS or Sβ^0^ thalassemia) with a hemoglobin electrophoresis in our system showing >50% hemoglobin S (or less in the setting of documented transfusions) who were ≥18years of age and who were seen at our institution between 1/1/2002 and 12/31/2002 were the inception cohort. Further analysis of the individual hb electrophoresis of these patients was then performed to exclude SC and **S**^**+**^ thalassemia. Patients were then subdivided into those who had died before 12/31/2012 and those who survived. Patients were excluded if documentation was not available to place patients in either category or they were lost to follow-up.

### Laboratory Determinants

Steady state laboratory tests were defined as those not within a day of an ED visit or a week of a hospital admission. All values obtained for 2002 were averaged: Hb, red blood cells (RBC), hematocrit (Hct), mean cell volume (MCV), mean cell hemoglobin concentration (MCHC), absolute reticulocyte count (Retic), platelets (Plt), white blood cell count (WBC), serum creatinine (Cr), albumin (Alb), total protein (Tprot), alkaline phosphatase, serum alanine transaminase, serum aspartate aminotransferase (AlkPhos, ALT, AST respectively), indirect bilirubin (IB), lactate dehydrogenase (LDH), %HbF. For the survival group, all 2012 steady state laboratory values were averaged. For the mortality group, the last steady state values before death in our system were obtained. Hemoglobin (Hb) electrophoresis was measured by high performance liquid chromatography. HbF and weight were not delineated by steady state.

### Clinical Data

The total number of Emergency Department visits, inpatient admissions, and clinic visits was recorded for each patient within 1/1/2002 to 12/31/2002 and within 1/1/2012 to 12/31/2012; for our mortality population, utilization in the last 12 months of their life was obtained. Hydroxyurea use was defined as positive if the patient was given one or more prescriptions for hydroxyurea within 1/1/2002 until 12/31/2002 and within 1/1/2012 until 12/31/2012. We were unable to obtain 2002 hydroxyurea use for our entire study sample; however data for a subset were obtained. Weight was defined as the first recorded weight measurement from 1/1/2002 until 12/31/2002 and from 1/1/2012 until 12/31/2012 or the last measurement before death.

### Statistical Analysis

Data were entered into Excel and transferred to SPSS. Data were analyzed for normality; parametric continuous values were summarized with means ±SD, nonparametric values were summarized as medians and interquartile ranges. Parametric and nonparametric bivariate tests of association were used as appropriate. Each measure of interest was compared separately and an alpha of .05 was used to assess for statistical significance. Data from 2002 and 2012 or last year before death were compared with paired-t tests. Survival between groups was compared using Kaplan-Meier curves and multivariable adjusted hazard rates were estimated with Cox proportional hazard models that included adjustments for age.

## Results

### Baseline (2002) Characteristics

**Fig 1:**
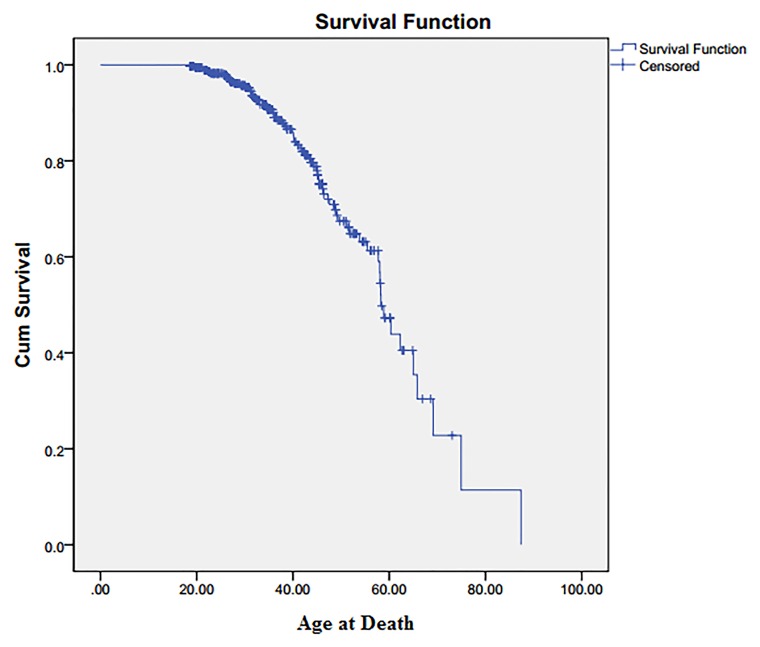
Kaplan Meier estimator for the study population. Median survival = 58.3 years.

We identified 289 SS/Sβ^0^ patients who had been present in our hospital system in 2002 and were still present in 2012. The average age for the survivor group in 2002 was 24.4 ±11.7yrs (range 7–62 years, 54% female). We also identified 70 patients who were present in 2002 but who had died between 2002 and 2012. Although the %female was somewhat lower at 47%, this was not significant. The average age of the mortality cohort was significantly different at 36.9 ±14.2yrs (p<0.001). Since the mortality group was, as expected, significantly older than the survivor group in the 2002 dataset, analyses were performed after age-adjustment. Average age at death was 42.1±14.0 years, median survival was 58.3 years (95% CI: 54.5, 63.0) (Fig 1). There was no statistically significant difference between the groups in prescriptions written for hydroxyurea. In 2012, 38.1% of the survival group were given a prescription for hydroxyurea compared to 36.1% in 2002.

### Mortality Risk for Baseline Laboratory Markers and Hospital Utilization

We first investigated whether our baseline predictors of mortality were similar to those demonstrated previously [1,3,6–8]. As shown in Table 1, Cox hazard ratios (HR), adjusted for age, were calculated to estimate relative risk of death over the decade (Table 1). Each 10^9^/L increase in steady state WBC was associated with a 12% greater mortality (p = .02); each mg/dL Cr was associated with a 43% greater mortality (p < .001); each 1% increase in HbF levels was associated with an 8% reduction in mortality (p = .001). Each 20 U/L increase in Alkaline Phosphatase and each 20 U/L increase in AST were associated with a 10% and 40% increase in mortality respectively. Other parameters of liver function such as total protein or ALT or other parameters of hemolysis such as LDH and indirect bilirubin were not significantly associated with changes in the mortality rate. Weight and sex showed no significant association with mortality risk; females did not live longer than males in our cohorts. When use of the emergency department (ED), clinic, and inpatient admissions were examined, only hospital admissions were associated with a 37% increase in mortality risk (p < .001).

**Table 1:** Age-adjusted Cox Hazard Ratios for Mortality.

	Cox Hazard(95% CI)	p
**HbF (%)**	0.92(0.88–0.97)	**0.001**
Hb (g/dL)*	0.80(0.61–1.00)	0.053
**MCV (g/dL)**	1.01(0.98–1.05)	0.5
**MCHC (g/dL)**	0.82(0.63–1.01)	0.06
**Reticulocyte Count (x10^9/L)**	1.00(0.99–1.00)	0.7
**Platelet Count (x10^9/L)**	1.00(1.00–1.00)	0.99
**WBC (x10^9/L)**	1.12(1.02–1.22)	**0.02**
**LDH (U/L)**	1.00(1.00–1.00)	0.6
**Cr (mg/dL)**	1.43(1.18–1.75)	**<.001**
**Albumin (g/dL)**	0.41(0.17–1.01)	0.051
**Alkaline Phosphatase (20 U/L)**	1.10(1.06–1.15)	**0.002**
**AST (20 U/L)**	1.40(1.04–2.14)	**0.02**
**ALT (U/L)**	1.02(0.99–1.04)	0.2
**Indirect bilirubin (mg/dL)**	0.94(0.81–1.09)	0.4
**ED Visits (/year)**	0.94(0.87–1.03)	0.2
**Clinic Visits (/year)**	1.01(0.99–1.03)	0.43
**Hospital Admissions (/year)**	1.37(1.26–1.48)	**<.001**
**Weight (KG)**	1.00(0.99–1.02)	0.7
**Male**	1.33(0.82–2.16)	0.2

*When adjusted for Creatinine, the p value of Hb = .07.

### Changes in Laboratory Markers over Time

When comparing longitudinal changes (Δ) in these parameters for those who survived the next 10 years (Table 2), there was a significant decrease in Hb (Δ-0.3g/dl, p = .01), %HbF (Δ-6.1%, p < .001), WBC (Δ-0.8 x10^9^/L, p = .01) and platelet count (Δ-32.3x10^9^/L, p = .003). Over these same 10 years, absolute reticulocyte counts increased (Δ+65.3x10^9^/L, p < .001) as did MCV (Δ+3.1fl, p < .001), while MCHC decreased (Δ-0.9g/dL, p < .001). There was a decrease in indirect bilirubin (Δ-0.6mg/dL, p = .003). Patients developed a mild but significant decrease in renal function (Δ+0.1mg/dL creatinine, p < .001).

**Table 2:** Laboratory and clinical trends over time for all patients in the mortality and survivor cohorts. Comparisons are made for each group separately and data are displayed as means ±SD for normal distributions and medians and interquartile ranges (/) for non-normal distributions.

Cohort	Survivor			Mortality		
Year	2002	2012	p	2002	Last Value	P
**HbF (%)**	12.3 (4.5/18.8)	6.2 (3.2/12.0)	**<.001**	6.1 (1.9/12.5)	8.6 (3.5/16.0)	0.7
Hb (g/dL)*	8.4 ±1.4	8.1 ±1.5	**0.01**	8.1±1.4	8.4 ±1.9	0.2
**MCV (g/dL)**	88.4 ±9.3	91.5 ±11.8	**<.001**	88.0±12.4	90.4 ±12.3	0.4
**MCHC (g/dL)**	35.1 ±1.2	34.2 ±1.3	**<.001**	34.4±1.9	33.5 ±1.7	**0.02**
**Reticulocyte Count (x10^9/L)**	182.4 (129.1/234.0)	247.4 (168.1/341.2)	**<.001**	183.4 (117.7/221.9)	173.9 (84.1/276.8)	0.7
**Platelet Count (x10^9/L)**	394.0 ±128.3	361.7 ±125.2	**0.003**	380.8±115.2	318.9 ±122.4	0.06
**WBC (x10^9/L)**	11.6 ±3.5	10.8 ±4.1	**0.014**	12.5±4.7	14.4 ±6.9	0.1
**LDH (U/L)**	404.0 (359.0/657.8)	397.0 (309.5/532.7)	0.6	423.5 (350.0/752.6)	417.0 (286.0/568.0)	0.7
**Cr (mg/dL)**	0.6 (0.5/0.7)	0.7 (0.6/0.9)	**<.001**	0.8 (0.6/1.4)	1.0 (0.7/1.9)	0.007
**Albumin (g/dL)**	4.3 ±.3	4.2 ±.5	0.053	4.1±.4	3.9 ±.8	0.1
**Total Protein (g/dl)**	7.8 ±0.5	7.6 ±0.7	**<.001**	7.8±.6	7.6 ±1.2	0.2
**Alkaline Phosphatase (20 U/L)**	109.4 (88.0/164.0)	91.3 (70.0/125.1)	**<.001**	121.0 (97.0/167.1)	117.0 (84.0/212.0)	0.6
**AST (20 U/L)**	39.0 (31.4/51.6)	37.2 (28.8/50.9)	0.2	39.6 (34.0/67.0)	41.0 (26.0/74.0)	0.2
**ALT (U/L)**	20.6 (14.0/29.0)	21.1 (16.0/31.4)	**0.02**	24.7 (15.0/32.9)	22.0 (15.0/36.0)	0.6
**Indirect bilirubin (mg/dL)**	2.5 (1.7/4.1)	1.9 (1.1/3.3)	**.003**	1.7 (0.9/3.5)	1.5(0.8/3.2)	0.5
**ED Visits (/year)**	2.6 ±3.9	5.2 ±10.7	**<.001**	1.9±3.3	1.3 ±2.3	0.3
**Clinic Visits (/year)**	6.8 ±8.3	7.3 ±10.5	0.5	8.3±11.5	7.4 ±9.5	0.6
**Hospital Admissions (/year)**	0.3 ±0.8	2.0 ±3.1	**<.001**	2±2.5	3.3 ±3.8	**0.006**
**Weight (kg)**	58.4 ±21.1	66.0 ±13.9	<.001	64.1±15.6	62.6 ±13.5	0.4

When the survivor population was separated into subsets < 30 years and ≥30 years of age, the younger population showed the larger decreases in %HbF (Δ-13.4%, p = .002), Alkaline Phosphatase (Δ-29.3 U/L p = .04), and MCHC (Δ-0.5g/dL p = .04). All other measured parameters were similar in magnitude and direction in the older and younger survivors.

We then considered whether the trajectory of those who had died within those 10 years were different. The average date of death in this group was 4.7 years from their last 2002 steady state data so one might have expected any changes to be less robust. However, when we analyzed their changes in laboratory parameters, their changes were the same or greater within the shorter time frame. This “mortality” group decreased their MCHC by Δ-0.9g/dl, increased their serum creatinine levels (Δ+0.2mg/dL, p = 0.007) and decreased their platelet counts (Δ-61.9 x10^9^/L, p = 0.06) within approximately half the time as those who survived 10 years.

Major differences were seen in the trend of WBC and Hb. When the direction and degree of change of these parameters was compared between the survivor and mortality groups (Table 3), both Hb and WBC demonstrated not only significant changes in survivor vs. mortality cohorts but these changes were in opposite directions. Survivors decreased their Hb over time while those that died in the interim actually increased their Hb levels (p = .04). Similarly, WBC count decreased in the survivor group while the mortality group experienced an increase in their steady state WBC (p = .05). Both groups decreased the %HbF over time but the survivor group, which began with a much higher %HbF, actually had greater decreases over time (p = .002).

**Table 3:** Longitudinal change between those who died (mortality cohort) and those who survived (survivor cohort) using patient matched data comparing the same patient averages from 2002 to 2012 or last data available (mortality cohort).

Cohort	Survivor	Mortality	Δ p
**N**	133	31	
**HbF (%)**	-7.2 ±12.6	-0.09 ±5.5	**0.002**
Hb (g/dL)*	-0.3 ±1.2	0.3 ±1.3	**0.04**
**MCV (g/dL)**	3.1 ±9.2	2.5 ±15.0	0.8
**MCHC (g/dL)**	-0.9 ±1.3	-0.9 ±1.9	1.0
**Reticulocyte Count (x10^9/L)**	95.4 ±138.3	9.3 ±178.7	0.1
**Platelet Count (x10^9/L)**	-30.3 ±123.1	-63.3 ±116.3	0.3
**WBC (x10^9/L)**	-0.8 ±3.7	1.9 ±6.8	**0.05**
**LDH (U/L)**	-21.5 ±324.2	74.4 ±310.2	0.6
**Cr (mg/dL)**	0.4 ±1.3	0.7 ±1.3	0.2
**Albumin (g/dL)**	-0.1 ±0.5	-.2 ±0.8	0.3
**Total Protein (g/dl)**	-0.2 ±0.7	-0.2 ±0.9	1.0
**Alkaline Phosphatase (20 U/L)**	-23.8 ±74.7	7.1 ±97.1	0.1
**AST (20 U/L)**	-2.1 ±49.4	7.9 ±31.0	0.2
**ALT (U/L)**	4.2 ±42.3	7.3 ±33.8	0.7
**Indirect bilirubin (mg/dL)**	-0.8 ±1.4	.4 ±3.0	0.6
**ED Visits (/year)**	2.6 ±11.1	-0.6 ±4.1	**<.001**
**Clinic Visits (/year)**	0.5 ±13.3	-0.9 ±13.7	0.4
**Hospital Admissions (/year)**	1.7 ±3.0	1.3 ±3.8	0.4
**Weight (kg)**	7.6 ±18.2	1.9 ±19.1	0.3

* = When those <30 years of age or ≥30 years of age in the survivor group were compared, the younger cohort had greater changes but were in the same direction.

As our mortality cohort was significantly older than our survivor cohort, models were age adjusted. We compared the deltas seen in our survivor cohort stratified by age (<30 or ≥30yrs) in order to assess if these degrees of change (Δ) were age-related differences rather than survivor/mortality differences [9, 10]. We also adjusted for the shorter duration of follow up for the mortality group. Those laboratory values that showed some differences in change over time between the survivor and mortality groups in the initial analysis were entered into a Cox model which included age and follow-up. Multivariable HRs were assessed for each (Table 4). When the Δ%HbF was adjusted for age and follow-up, we could no longer detect a significant association with mortality. However, the increase in WBC (HR 1.16, 95% CI 1.04, 1.30; p = .01) and the increase in Hb level (HR 1.54, 95% CI 1.08, 2.18; p = 0.02) remained very significant. An increase in WBC over 10 years had 54.0% sensitivity and 84.6% specificity for risk of death, and had a 94.4% negative predictive value. An increase in Hb over 10 years had 61.5% sensitivity and 59.0% specificity, and had an 87.8% negative predictive value.

**Table 4:** Mortality Risk of Longitudinal Changes in Laboratory Parameters: Adjusted Hazard Ratios.

Cohort	Hazard Ratio	p
**N**	164	
**HbF (%)**	1.05 (0.99–1.12)	0.1
**Hb (g/dL)**	1.54 (1.08–2.18)	**0.02**
**Reticulocytes (x10^9/L)**	1.00 (1.00–1.00)	0.2
**WBC (x10^9/L)**	1.16 (1.04–1.30)	**0.01**
**Cr (mg/dL)**	1.12 (0.83–1.51)	0.5
ED Visits (/year)*	.84 (0.73–0.96)	**0.01**

### Longitudinal Assessments of Clinic and Hospital Utilization

When markers of clinical utilization were compared between 2002 and 2012 in the survivor group, there was increased use of the emergency department (2.6 visits increasing to 5.2 visits over 10 years, p < .001) (Table 2) and increased admission frequency (0.3 admissions increasing to 2.1 admissions per year, p < .001) but no significant change in number of clinic visits per year (6.8 vs. 7.3 visits, p = 0.50). The mortality group, in the last 12 months before death, also showed an increase in their admission frequency over baseline (2.0 vs. 3.3 admissions/yr., p = .006) and no meaningful change in clinic use (8.3 vs. 7.4 visits, p = 0.60) but the ED visits for those that died was actually lower than for those that survived. When the changes between the two groups were compared (Table 3), the survivor group showed a greater increase in emergency room use than the mortality group (Δ+2.6 vs. Δ-0.6 respectively). Table 4 demonstrates that the increase in emergency department visits actually significantly decreased age-adjusted mortality hazard by 16% (p = 0.01, Table 4).

## Discussion

As bone marrow transplantation becomes more prevalent, it becomes more important to find markers that can aid in risk stratification for morbidity and mortality in sickle cell disease [4, 5, 11, 12]. Previously, steady state biomarkers have been examined and %HbF, WBC, Creatinine, pain crises, and hydroxyurea use have generally been accepted to be markers for survival [1, 2, 13–17]. What is not clear is whether changes in these biomarkers over time in any one individual also have prognostic value; i.e. when we first see the patient with a WBC of 8.0 x10^9^/L, is there significance if the WBC rises to 8.8 x10^9^/L vs. if the WBC remains the same. Longitudinal studies allow us not only to better understand the natural history of sickle cell disease, but to attempt to predict the natural history so as to know when more aggressive intervention is warranted [18]. This is the first study to examine these longitudinal changes in steady state markers and the association of these changes on mortality risk. By examining patient matched data over time, we demonstrate here an association of increases in WBC and Hb over time with increased mortality.

Though a high WBC has been previously shown to be associated with early mortality, observed again in this study, our data suggest that increases in WBC over time is also a risk factor [1, 2]. We were unable to access death certificates to assess cause of death in our own cohort, but previous studies which were able to do so have shown that in the last two decades adults with sickle cell disease are most likely to die of pulmonary hypertension, sudden death, renal failure, infection, thromboembolism, cardiac diseases, cirrhosis, and acute chest in descending order [19]. WBC has been repeatedly demonstrated to correlate with stroke, pulmonary hypertension, and acute chest, as well as the frequency of pain crises [20–23]. We therefore postulate that, in addition to a high baseline WBC count, an increasing WBC over time is an independent risk factor for mortality.

Some studies have shown low hemoglobin to also be a risk factor for early mortality in SCD patients, but others have disagreed [1, 2, 6]. The 9-year follow up of hydroxyurea in SCA patients showed that anemia was a risk factor for mortality only in the setting of low absolute reticulocyte counts, and a more recent study which conflated all types of SCD observed that while anemia had a significant association with mortality, it lost this association when hemoglobin was adjusted for renal failure [8, 17]. Our study did not observe that baseline anemia or an increase in anemia over time, particularly when adjusted for renal failure, was a significant risk factor for mortality. Even more surprisingly our data suggest that not only may there be no association of low Hb with a higher mortality, but that an increase in hemoglobin over time may increase mortality risk. Previous studies of aging and the elderly in sickle cell disease have shown that Hb may be expected to decrease over time.[17, 24–27] Our survivor population did show this expected decrease; indeed our mortality population suggest that the lack of this decrease might be a cause of concern in the long term follow-up of the individual patient. In support of this, the Cooperative Study of Sickle Cell Disease showed that SCA patients with *higher* hematocrits were more likely to experience more episodes of pain crisis, and that more pain crisis were associated with greater mortality risk [13]. Since both higher WBC and higher Hb levels contribute to greater vasoocclusion, these values are potentially useful markers for mortality and continued longitudinal assessment may be important for ultimate prognosis as well as for risk stratification to identify patients in need of more aggressive therapy [6, 10].

When health care utilization was examined, both groups showed no change over time in their clinic use and both showed a similar level of increasing admissions. Previous studies of ED use have shown mixed results on whether the aging sickle cell population utilizes the ED more or less often [7, 28, 29]. Our survivor group showed an increase in ED use while the mortality cohort did not. It may be that the survivor group seeks and receives immediate medical treatment earlier, thereby eliminating the need for admission, while the mortality group may be more likely to present when they are substantially sicker and require admission. It may also be that our mortality cohort, with their greater number of annual admissions, spends more time in the hospital as an inpatient and thus have less time as an outpatient where they may present to the emergency room for care.

Our study has important contribution and important limitations. It is the largest study to follow individual adult SCA patients but it is a retrospective study, which can create possible bias and limit our ability to obtain certain steady state markers. We lacked autopsy data to allow us to evaluate cause of death in our mortality group, thus we were only able to evaluate risk of overall mortality and not cause specific mortality. We collected steady state data and we lack data on specific acute clinical complications (i.e. stoke, acute chest, pain crisis) experienced by our cohort. Our study, therefore, assesses the effects of our longitudinal markers on all-cause mortality; further studies are needed to examine the correlation between longitudinal changes in biologic markers with the development of the clinical sequalae of sickle cell disease.

No correction for multiple testing was applied but the interpretation of results was with awareness of potential inflated type 1 error. Although we were unable to obtain all 2002 data on hydroxyurea use, we were able to obtain from a subset which showed that a similar percentage of sickle cell patients were given hydroxyurea prescriptions both in 2002 and 2012 (36% vs 38%) so this is unlikely to account for variations seen over time.

Finally, we did not evaluate our group for the presence of alpha thalassemia. Though some studies have suggested that patients with SCA and concomitant alpha thalassemia have decreased mortality rates, larger cohort studies have failed to bear out these findings [2, 30, 31].

In summary, we demonstrate that increases in WBC and Hb over time are markers for earlier mortality. In addition, and paradoxically, increased emergency department visits over time were associated with decreased mortality. We suggest that assessing steady state values and monitoring their change over time may be an improved means of assessing survival in SCA. This may have important ramifications in disease stratification for therapeutic interventions, particularly for those interventions that have significant morbidity/mortality such as bone marrow transplant. Knowing *when* to be therapeutically aggressive as part of the risk:benefit analysis may be of vital importance when making life-changing decisions on individual patients.

## Supporting Information

S1 File The original laboratory and clinical data has been attached as an excel spreadsheet. It is available upon request.
(XLS)
